# 1084. Comparative Outcomes Among Patients Receiving Varying Daptomycin Dosing Regimens in Hemodialysis

**DOI:** 10.1093/ofid/ofab466.1278

**Published:** 2021-12-04

**Authors:** Tyler Maxwell, James E Orban, Wesley D Kufel, Christopher Destache, Karen S Williams, Manasa Velagapudi, P Brandon Bookstaver, Luis Tatem, James A McCracken, Bethanne Carpenter, Roopali Sharma

**Affiliations:** 1 SUNY Downstate Medical Center, Brooklyn, New York; 2 University of South Carolina, Columbia, South Carolina; 3 Binghamton University School of Pharmacy and Pharmaceutical Sciences, Binghamton, New York; 4 Creighton University Schools of Medicine and Pharmacy, MDstewardship, Omaha, Nebraska; 5 Guthrie Robert Packer Hospital, Sayre, Pennsylvania; 6 CHI Health - Creighton University Medical Center - Bergan Mercy, Omaha, Nebraska; 7 University of South Carolina College of Pharmacy, Columbia, SC; 8 Rutgers, The State University of New Jersey, Asbury Park, New Jersey; 9 County of Santa Clara Health System, San Jose, California; 10 Touro College of Pharmacy, New York, NY

## Abstract

**Background:**

Daptomycin (DAP) has become an appealing treatment option for gram-positive infections, which are common in patients receiving hemodialysis (HD), due to frequent access and manipulation. The approved DAP dosing of 4 to 6 mg/kg every 48 hours (q48h) quickly becomes desynchronized from the patient’s HD schedule and requires the burden of additional IV access. Previous pharmacokinetic studies have suggested that DAP can be dosed three-times weekly following HD, but no studies have evaluated clinical outcomes of this regimen.

**Methods:**

This was a multi-center, retrospective cohort study across 6 hospitals in the United States. Adult, nonpregnant patients who received HD and DAP between 2015 and 2020 were screened for inclusion. Electronic medical records were reviewed for relevant study data. The primary outcome was clinical and microbiological outcomes among patients who received DAP thrice weekly versus q48h dosing. Microbiological Failure was defined as positive cultures after 7 days and further study definitions are included under Table 3.

**Results:**

Baseline characteristics are summarized in Table 1. Length of stay was similar between both groups at a median of 25 days and patients had a median QPitt score of 0 on admission. The average DAP dose used was 7 mg/kg and 7mg-7mg-9mg on HD days in the q48h dosing and thrice weekly dosing regimens, respectively. The majority of patients had bacteremia and the most commonly isolated bacteria was methicillin-resistant *Staphylococcus aureus*. No differences in clinical outcomes were observed (p=0.87). Microbiological failure was higher among patients who received DAP thrice weekly compared to q48h dosing (69.2% vs 34.8%, p=0.047).

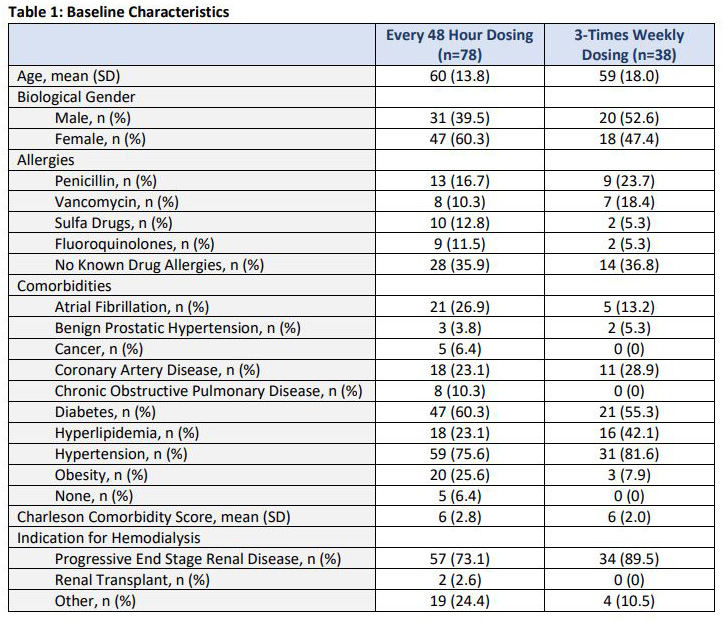

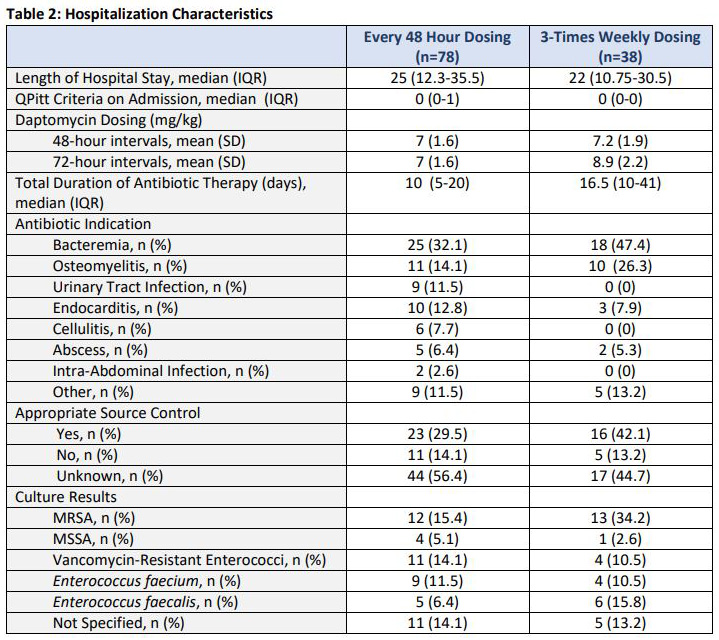

**Conclusion:**

DAP dosed thrice weekly on HD days offers similar clinical resolution compared to q48h dosing. While the thrice weekly dosing regimen did have a significantly higher rate of microbiological failure, the analysis was limited by a small sample size. As this is a retrospective analysis not accounting for confounding variables, additional prospective studies are warranted to confirm these findings.

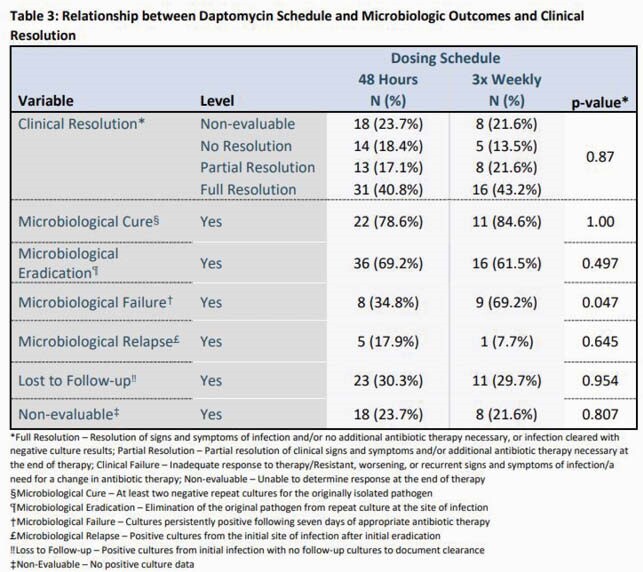

**Disclosures:**

**Wesley D. Kufel, PharmD**, **Melinta** (Research Grant or Support)**Merck** (Research Grant or Support)**Theratechnologies, Inc.** (Advisor or Review Panel member) **P. Brandon Bookstaver, Pharm D**, **ALK Abello, Inc.** (Grant/Research Support, Advisor or Review Panel member)**Biomerieux** (Speaker's Bureau)**Kedrion Biopharma** (Grant/Research Support, Advisor or Review Panel member)

